# Vascularization in Biofabrication: Innovative Strategies for Advanced Tissue Models

**DOI:** 10.3390/cells15141245

**Published:** 2026-07-10

**Authors:** Yvonne Kulicke, Rafael Schmid, Theresa Promny, Tannaz Karimi, Evelin Sandor, Lisa Wedler, Celena Soergel, Andreas Arkudas, Annika Kengelbach-Weigand, Raymund E. Horch

**Affiliations:** Laboratory for Tissue Engineering and Regenerative Medicine, Department of Plastic and Hand Surgery, University Hospital Erlangen, Friedrich-Alexander-Universität Erlangen-Nürnberg (FAU), D-91054 Erlangen, Germanytheresa.promny@uk-erlangen.de (T.P.);

**Keywords:** biomaterials, bioprinting, vascularization, 3D tissue models

## Abstract

The increasing demand for tissue and organ replacement has positioned biofabrication as a transformative field within regenerative medicine. Biofabrication is an interdisciplinary approach that combines cells, biomaterials, and bioactive molecules to generate biologically functioning structures and tissue models. However, the size of biofabricated constructs often remains limited due to the lack of vascular structures. Cells located in the inner regions of larger constructs suffer from a lack of oxygen and nutrients, which results in hypoxia and reduced cell viability. Furthermore, integration into the host vasculature is crucial for the long-term survival of constructs intended for in vivo implantation. Consequently, strategies to improve the vascularization of biofabricated constructs have gained significant attention. In this review, we focus on approaches for the in vitro and in vivo vascularization of biofabricated constructs. We summarize the types and sources of vascular cells used in biofabrication, as well as the signaling molecules and biomaterials that can be incorporated into biofabricated scaffolds. A special focus is placed on advanced vascularization strategies such as the *in ovo* chorioallantoic membrane (CAM) and in vivo arteriovenous (AV) loop models. Furthermore, we describe the role of vascularization in advanced tissue models used for disease modeling and drug screening. Although the clinical translation of biofabricated constructs is still limited by a lack of vasculature and size constraints, biofabrication offers great potential for tissue replacement, personalized medicine, and the reduction of animal experiments.

## 1. Introduction

Discoveries and breakthroughs in science in recent decades have led to a rapid increase in patient life expectancy. Alongside this increase, a surge in the demand for tissue and organ transplants can be observed [1]. To treat end-stage organ failure, the transplantation of organs from living or deceased donors often remains the only option. However, the demand for transplanted organs far exceeds the supply, and patients still face challenges such as organ rejection or complications associated with immunosuppression [2].

The research fields of biofabrication and tissue engineering aim to fabricate tissue and organ substitutes through a combination of biomaterials, cells and engineering approaches. These substitute tissues may be utilized to replace damaged or diseased tissue. At the same time, they can be used as advanced in vitro models to study both healthy and diseased tissues. These models can reduce the number of animals used in research and enable the use of human and even patient-derived cells. Consequently, they can more accurately reflect the clinical situation and improve clinical translation.

Tissue engineering typically combines cells, biomaterials, and bioactive molecules to restore, maintain, or improve the function of damaged or diseased tissues and organs. The conventional method of tissue engineering involves seeding patient-derived cells onto a variety of biomaterials, so-called scaffolds [3,4]. Biofabrication, on the other hand, refers to the specific distribution of cells, biomaterials and biologically active factors on 3D scaffolds or in hydrogels, which are spatially arranged during production to ultimately generate a biologically functional product [5,6]. While tissue engineering represents the broader regenerative medicine concept, biofabrication specifically emphasizes the controlled fabrication and spatial distribution of living cells and biomaterials. The precise arrangement of the components is often achieved via bioprinting [5,7,8]. Bioprinting is a rapid, cost-effective technology that facilitates the structured arrangement of cells and enables the construction of complex tissue architectures, which can be shared easily among researchers and medical professionals [9,10,11].

However, despite significant technological progress, the size of biofabricated constructs remains limited due to a lack of vascularization. Oxygen diffusion in living tissue is limited to about 100–200 µm, making passive diffusion inadequate for larger constructs [12]. As a consequence, cells located in the inner parts of large constructs often suffer from an insufficient oxygen and nutrient supply, leading to hypoxia, reduced cell viability, and necrosis. For clinical-sized constructs intended for in vivo implantation, rapid integration into the host vasculature is essential to ensure long-term survival, functionality, and successful tissue regeneration. The development of vascularized tissue thus remains one of the major challenges in biofabrication [13]. Common strategies to introduce a vascular component are the 3D printing of tubular structures or the incorporation of umbilical vein endothelial cells, which can support the formation of primitive vascular networks and improve oxygen and nutrient diffusion. However, they only partially recapitulate the complexity of the in vivo microenvironment, particularly regarding the specialized phenotypes and tissue-specific functions of endothelial cells. Consequently, there is an increasing interest in advanced vascularization strategies that more closely mimic physiological conditions. In this context, special attention will be given to models such as the chorioallantoic membrane (CAM) model or the arteriovenous (AV) loop model, which provide dynamic and highly vascularized environments for studying tissue integration, angiogenesis, and functional vascular network formation in biofabricated constructs.

This review presents an exploration of the evolving field of vascularization in biofabricated constructs. First, we provide an overview of the most commonly used cell types, signaling molecules, and biomaterials. Next, we describe the main strategies for incorporating a vascular component into biofabricated models, as well as the main types of assays to assess angiogenesis in vitro. We place a special focus on advanced in vivo approaches, such as the chorioallantoic membrane (CAM) model and the arteriovenous (AV) loop model. Furthermore, we describe common applications of vascularized biofabricated constructs, such as advanced tissue models, drug screening platforms, and personalized medicine. Lastly, we set out the future prospects for the field of vascularization in biofabrication, including the potential for advancements in technology and applications.

A literature search was conducted using the PubMed database. Publications were identified using keywords such as *biofabrication*, *vascularization*, *vascular cells*, *bioprinting*, *angiogenesis*, and *tissue models*. This review primarily considered primary research articles published between 2015 and 2026, with systematic and narrative reviews as well a s earlier publications included when necessary to describe fundamental concepts.

## 2. Vascularization Strategies for Biofabricated Constructs

**Figure 1:**
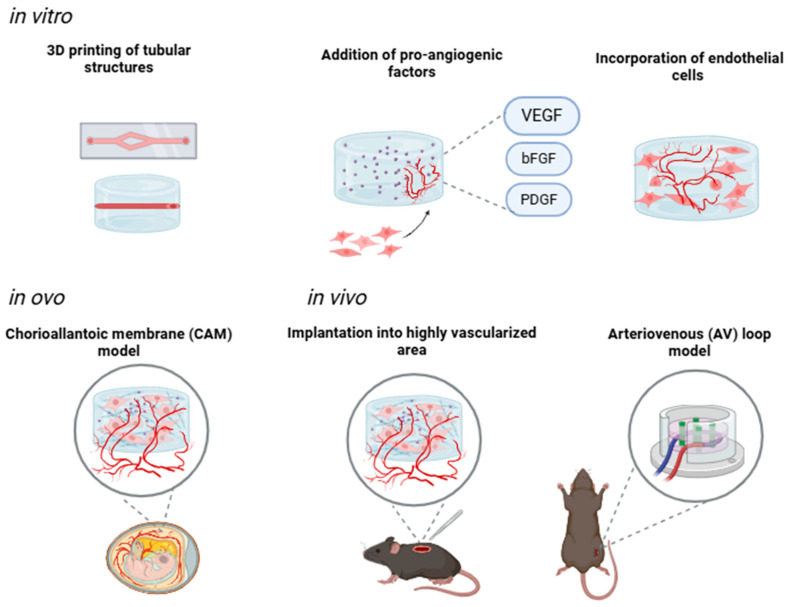
Vascularization strategies in biofabrication. In vitro, *in ovo* and in vivo approaches can improve the vascularization of biofabricated constructs. In vitro, 3D printing can be used to generate tubular structures resembling blood vessels, which improve diffusion of oxygen and nutrients. The sprouting, migration and proliferation of incorporated vascular cells can be improved by the addition of pro-angiogenic factors such as VEGF, bFGF and PDGF. The chorioallantoic membrane (CAM) is a highly vascularized membrane in the developing avian egg, which can be utilized to analyze angiogenic properties of biomaterials. In vivo, vascularization can be facilitated by implantation of the constructs into highly vascularized areas of the body, or by introducing axial vascularization by means of an arteriovenous loop. Created in BioRender. Sandor, E. (2026) https://BioRender.com/beojwut (accessed on 5 July 2026).

Sufficient oxygen and nutrient supply is critical for cell survival in biofabricated constructs. In the human body, blood vessels transport blood cells, oxygen and nutrients and they play a crucial role in organ and tissue homeostasis. Replicating the functions of the vascular network is essential for more complex biofabricated models. However, as the limit for the efficient diffusion of oxygen is 100–200 µm, cells in larger constructs often experience hypoxia and limited access to nutrients, leading to necrosis and reduced functionality [12]. Thus, the efficient vascularization of larger constructs must be achieved. The main strategies to improve vascularization of biofabricated constructs are summarized in Figure 1, while their advantages and disadvantages are compared in Table 1.

Many previous works have only included a limited vascular component, such as simple tubular structures or an immortalized form of human umbilical vein endothelial cells (HUVECs). Although these strategies can improve oxygen and nutrient supply, they fail to recapitulate the complex vascular microenvironment in vivo. The native vasculature differs not only between organs but also by developmental stage. Furthermore, blood vessels consist of several cell types such as endothelial cells, smooth muscle cells, and pericytes, all of which contribute to the correct function of the vascular system and must be taken into account when establishing biofabricated constructs.

In the developing embryo, blood vessels arise via two distinct processes referred to as vasculogenesis and angiogenesis. Vasculogenesis is the de novo formation of blood vessels from mesoderm-derived precursor cells [14]. During early embryonic development, the mesodermal layer is formed between the endoderm and the ectoderm in a process called gastrulation. Mesodermal precursor cells differentiate into endothelial progenitor cells, which in turn form the first vascular structures. Subsequently, new blood vessels form through the sprouting, splitting or fusing of existing vessels, which is known as angiogenesis [15].

There are two types of angiogenesis: sprouting and intussusceptive angiogenesis. Sprouting angiogenesis is the primary process through which new blood vessels arise in the mature human body [16,17]. Upon stimulation via pro-angiogenic factors such as vascular endothelial growth factor (VEGF), endothelial cells in the vessel lining start to proliferate and migrate. One cell is designated as the tip cell, which sprouts outwards and migrates towards the pro-angiogenic signal. Following the tip cell, stalk cells proliferate and form a hollow tube which later creates the lumen of the newly formed vessel. A new vascular network is formed by the interconnection of the new tubular structures. Intussusceptive angiogenesis is also known as splitting angiogenesis. A single, pre-existing blood vessel divides into two separate vessels by folding inwards [18]. The two opposing walls of the capillary establish a zone of contact. The cell junctions between the endothelial cells then reorganize and the bilayer perforates, enabling cells to penetrate into the lumen. A tissue core is formed at the contact zone, which divides the original vessel into two distinct sections. Finally, the core is filled with extracellular matrix components, thereby completing the separation while maintaining the functional integrity of the vessels. Compared to sprouting angiogenesis, intussusceptive angiogenesis mainly focuses on the rearrangement of existing cells and is particularly relevant in embryonic development [17].

The stages of vasculogenesis and angiogenesis are regulated by the differential expression of a variety of transcription factors, which have been reviewed in detail by Payne et al. [14]. Additionally, angiogenesis and vascular homeostasis are regulated by metabolic and environmental factors such as hypoxia, mechanical cues, and glycometabolism [19,20,21]. Consequently, culture conditions have a major influence on vascular cell proliferation, migration and gene expression in vitro.

The most commonly used cell types for biofabrication, as well as their sources and functions, will be described in the next section.

### 2.1. Types and Sources of Vascular Cells

Blood vessels consist of three main cell types: endothelial cells form a selectively permeable barrier between the blood and the surrounding tissue, smooth muscle cells control vessel constriction, and pericytes maintain homeostasis and hemostasis [22]. The function of vascular cells varies not only by cell type, but also by their tissue of origin. The organotypic variety in the gene expression of these cell types was mapped by Barnett et al., who used single-cell transcriptomics data from 19 different human organs and tissues to identify 42 distinct types of vascular cells [23]. This variability needs to be taken into account when generating realistic in vitro tissue models. Although endothelial cells are the most common cell type incorporated in biofabricated constructs because of their wide availability and relative ease of cultivation, there is a growing number of advanced tissue models containing different types of vascular cells. For example, many models now contain organ-specific vascular cells to better recapitulate their native function.

The most commonly used cell types in biofabrication will be described in the following sections. Their main advantages and disadvantages are also compared in Table 2.

#### 2.1.1. Vascular Endothelial Cells

Vascular endothelial cells display high levels of heterogeneity and perform specialized roles throughout the body [24]. Arterial and venous endothelial cells differ not only in morphology but also in gene expression and the organization of cell–cell junctions: arterial endothelial cells are long, narrow, and form tighter intercellular junctions, while venous endothelial cells are rounder and show more loosely organized tight junctions [25]. The shape and function of endothelial cells also vary greatly between different tissues and organs. For example, the fenestrated endothelium in the kidney is highly permeable, which facilitates the removal of waste products and toxins [26]. In contrast to that, the endothelial cells in the blood–brain barrier form a highly selective structural barrier due to the presence of tight junctions, and they restrict the entry of blood-borne molecules into the brain [27]. Consequently, the source of endothelial cells applied in biofabrication must be taken into consideration to ensure the correct function of the resulting constructs. Due to organ-specific differences between endothelial cells, it is advisable to use endothelial cells from the respective target organ for tissue models. For example, renal endothelial cells should be used for biofabricated kidney models, while brain endothelial cells are best suited for models of the central nervous system [28,29]. The transcriptomic signatures of endothelial cells vary greatly by the tissue of origin but are also influenced by the sex of the donor, which should be taken into consideration [30].

Human umbilical vein endothelial cells (HUVECs) are commonly used because of their ease of isolation and culture, but they may not closely replicate the function of tissue-specific endothelial cells. The isolation of HUVECs was first described in 1973 by Jaffe et al. [31]. HUVECs are typically obtained via the enzymatic digestion of the umbilical vein. Primary HUVECs can undergo up to 65 population doublings in vitro [32]. However, since they display senescence-related changes in cell morphology and gene expression, it is advisable to use HUVECs in early passages. To prolong the lifespan of HUVECs, several immortalized cell lines have been established. The EA.hy926 cell line is a hybrid between HUVECs and a human lung carcinoma cell line [33]. EA.hy926 cells retain the gene expression profile of endothelial cells, but they also express a variety of genes that are not expressed in HUVECs [34]. Alternatively, HUVECs can be immortalized by the ectopic expression of the catalytic subunit of human telomerase (hTERT) [35]. These cells maintain the expression of many endothelial marker genes, but they still display differences in gene expression compared to primary cells. Consequently, the suitability of immortalized cells needs to be validated depending on the application.

#### 2.1.2. Induced Pluripotent Stem Cells

Endothelial cells can also be differentiated from human induced pluripotent stem cells (hiPSCs). iPSCs can be derived from adult somatic cells by reprogramming them into an embryonic stem cell-like state. In 2006, the team of Shinya Yamanaka demonstrated that adult mouse fibroblasts could be reverted to a pluripotent state by the overexpression of four transcription factors (Oct4, Sox2, Klf4 and c-Myc), the so-called “Yamanaka factors” [36]. Since then, various protocols have been established to induce pluripotent stem cells and differentiate them into a variety of cell types. Endothelial cells derived from hiPSCs (hiPSC-ECs) can also be further differentiated into arterial, venous, or organ-specific phenotypes [37]. However, the generation and differentiation of hiPSC-ECs is often labor-intensive and costly. It should be taken into account that the donor’s genetic background and epigenetic factors influence the differentiation of iPSCs. This was shown in an RNA sequencing analysis by Caarcamo-Orive et al., who compared gene expression in hiPSC lines from 101 individuals [38]. Biological variables such as donor age, sex and body mass index were shown to only affect a subset of genes, while the majority of gene expression variability is caused by genetic differences between individuals. This may lead to increased variation when hiPSC-ECs from different donors are used. However, patient-specific hiPSC-ECs are of great interest for disease modeling and personalized medicine. For example, Zhou et al. used patient-derived hiPSC-ECs to investigate the endothelial cell alterations in atypical hemolytic uremic syndrome [39]. The patient-derived hiPSC-ECs exhibit functional defects such as decreased migration and proliferation, and they display aberrant p38/MAPK signaling. Similarly, hiPSC-ECs from donors with type 2 diabetes mellitus show increased senescence, impaired mitochondrial function, and lower angiogenic potential [40].

#### 2.1.3. Endothelial Progenitor Cells

The term “endothelial progenitor cells” (EPCs) is commonly used to describe a subtype of progenitor cells that possess the capacity to differentiate into mature endothelial cells [41]. In vitro, EPCs can be differentiated via the addition of growth factors such as VEGF [42]. They were initially thought to originate from the bone marrow [43]. However, recent studies indicate that they reside in a vascular niche within the blood vessel wall [44,45]. They can be isolated from different sources, such as peripheral blood, bone marrow, umbilical vein blood, or adipose tissue [46,47]. EPCs are most commonly identified via cell surface markers such as CD34, VEGFR2, CD31, CD146, and CD144 [48]. Yet, due to a lack of standardization regarding EPC-specific markers, the identification and characterization of EPCs vary greatly between different studies, thus limiting reproducibility [49]. Due to their low abundance, the availability of primary EPCs is limited. Consequently, immortalized EPC cell lines such as HEPC-CB1 have been established [50,51].

#### 2.1.4. Mesenchymal Stem Cells

Mesenchymal stem cells (MSCs) can also be differentiated into endothelial cells [52]. MSCs can be isolated from various tissues such as bone marrow, adipose tissue, umbilical cord, dental pulp, or synovium [53]. They differentiate into endothelial cells when stimulated with VEGF, although other growth factors such as bFGF may be added as well [42,54]. However, they display high levels of heterogeneity depending on their source, isolation, and differentiation method [49].

#### 2.1.5. Vascular Smooth Muscle Cells

Vascular smooth muscle cells (VSMCs) are located in the media layer of blood vessels. They regulate blood flow, vessel diameter, and blood pressure via contraction and relaxation [55]. Primary VSMCs can be isolated via the enzymatic dissociation of large blood vessels, such as the aorta of mice or rats [56]. Human VSMCs can also be obtained from the umbilical cord, carotid atherosclerotic plaques, or the internal mammary artery (from patients undergoing bypass surgery) [57,58,59]. Alternatively, VSMCs can be differentiated from hiPSCs [60]. In biofabrication, VSMCs are an essential component of blood vessel models capable of contraction. Mastoor et al. established a hydrogel channel with circumferentially aligned VSMCs, which recapitulates their native alignment in blood vessels [61]. Similarly, Derhambakhsh et al. electrically stimulated VSMCs on a vascular graft to convert them into a contractile phenotype [62].

#### 2.1.6. Pericytes

Pericytes are specialized contractile cells that line the outer wall of capillaries and small blood vessels [63]. They are embedded in the basal membrane in close contact with endothelial cells, and they play a major role in angiogenesis, tissue regeneration and local control of capillary blood flow. In the central nervous system, they also play a crucial role in the blood–brain barrier [64]. Pericytes display functional and morphological heterogeneity depending on their tissue of origin and position along the capillary bed, which should be taken into account when selecting a protocol for cell isolation [65]. They can be harvested from a variety of tissues such as the brain, the heart, or the umbilical cord [66,67,68]. Pericytes are commonly identified by their expression of cell surface markers, which were reviewed in detail by Alvino et al. [69]. Alternatively, they can be differentiated from hiPSCs [70]. Because of their organ-specific functions, their use in biofabrication is not limited to vascular constructs. Jung et al. incorporated pericytes into a 3D model of the respiratory epithelium, while Seo et al. included pericytes in a model of the blood–brain barrier [71,72].

### 2.2. Incorporation of Pro-Angiogenic Signaling Molecules

In order to stimulate blood vessel growth and maturation, pro-angiogenic factors may be incorporated into biofabricated scaffolds. These can include growth factors, peptide tags, or bioactive glasses.

#### 2.2.1. Pro-Angiogenic Growth Factors

Scaffold materials can be supplemented with growth factors such as VEGF-A, bFGF or PDGF to enhance the proliferation and differentiation of the implanted cells. The advantages of incorporating growth factors into a hydrogel scaffold include a prolonged half-life, a reduction in negative effects such as burst release or unwanted diffusion, and the ability to spatially arrange them via 3D printing [73]. Alternatively, cells expressing signaling molecules can be incorporated into the constructs. Several cell types, such as vascular cells, osteoblasts or fibroblasts, naturally express pro-angiogenic growth factors [74,75,76]. The bioprinting process itself may enhance the expression of signaling factors by these cells [74]. Likewise, the hypoxia occurring in larger constructs is also a stimulus for the expression of pro-angiogenic growth factors [77]. Alternatively, cells can be transfected to overexpress pro-angiogenic factors either constitutively or regulated via a hypoxia-induced gene expressing system [78,79,80].

Vascular endothelial growth factor A (VEGF-A) is one of the best-characterized and most commonly incorporated pro-angiogenic growth factors. It is part of the VEGF family, which comprises VEGF-A, placenta growth factor (PGF), VEGF-B, VEGF-C and VEGF-D. Since VEGF-A was the first member of the family to be discovered and plays a dominant role in regulating angiogenesis, it is frequently referred to only as VEGF [81]. VEGF-A stimulates the proliferation, differentiation, migration and survival of endothelial cells [82]. Furthermore, it increases vascular permeability.

Basic fibroblast growth factor (bFGF), also known as FGF-2, is often combined with VEGF-A because of its synergistic effect [83,84]. Similar to VEGF-A, bFGF stimulates endothelial cell survival, proliferation and migration [85]. bFGF signaling also induces the secretion of ECM-remodeling enzymes such as matrix metalloproteases (MMPs), which play an important role in endothelial cell migration and sprouting [86].

Platelet-derived growth factors (PDGFs) are a family of growth factors that stimulate the proliferation of cells of mesenchymal origin, such as fibroblasts, pericytes, and smooth muscle cells [87]. During angiogenesis, they play an important role in vessel maturation and the recruitment of pericytes and smooth muscle cells [88,89]. PDGF may be delivered in combination with other growth factors such as VEGF-A and bFGF to enhance the maturation of newly formed vessels [90].

#### 2.2.2. Peptide Tags

Peptide epitopes of structural and signaling ECM proteins are used to design biomaterials for specific applications. This is particularly important for synthetic polymers or materials that are not derived from mammals, since they often lack adhesion sites or signaling motifs. A detailed library of functional epitopes has been compiled by Ligorio and Mata [91]. Among the most common epitopes is the RGD motif, which comprises the arginine–glycine–aspartic acid amino acid sequence. It is found on many ECM proteins such as fibronectin, collagen and osteopontin, and it serves as a recognition site for different integrins [92]. Since endothelial cells express high numbers of integrin receptors, the addition of the RGD motif to scaffold materials can improve vascularization of the constructs [93,94]. Other examples are the peptides QK and IQ, which mimic the growth factor VEGF-A [95,96]. In addition to naturally occurring motifs, cell adhesion peptides can also be identified via the high-throughput screening of peptide libraries. For example, Sonnentag et al. screened a library of over 11,000 peptide sequences to identify peptides with cell binding and cell repulsion properties [97].

#### 2.2.3. Bioactive Glasses

Bioactive glass particles are increasingly used as additives, in particular in the field of bone regeneration. The first bioactive glass, with the trademarked 45S5 Bioglass^®^, was developed in the late 1960s [98]. Since then, various compositions of bioactive glass have been established. They are able to stimulate specific cellular responses via the release of ions [99]. For example, silicon ions can stimulate the expression of VEGF and FGF by endothelial cells, while borate ions can increase cell proliferation by the activation of the MAPK signaling pathway [100,101].

## 3. In Vitro Vascularization Strategies

Due to their lack of vascular-like structures, large biofabricated constructs often have limited diffusion into their inner regions, which restricts the supply of oxygen and nutrients to the cells. Furthermore, constructs implanted in vivo often fail to integrate into the host’s vasculature, thus limiting the survival of the implanted cells. To address these challenges, various strategies can be applied, such as the addition of vascular cells, the creation of tubular structures via 3D bioprinting, or the incorporation of sacrificial materials to increase porosity.

### 3.1. Incorporation of Vascular Cells

To introduce a simple vascular component into biofabricated constructs, endothelial cells can be incorporated into the hydrogel matrix without further spatial arrangement [22]. The cells then self-assemble to form a microvascular network. However, due to a lack of signaling cues, these networks are typically unstable and poorly organized. The importance of the patterning and organization of endothelial cells in engineered constructs was previously highlighted in a review article by Rouwkema and Khademhosseini [102]. The incorporation of pro-angiogenic signaling molecules such as VEGF or angiopoietin-1 into the matrix can enhance cell proliferation and tube formation of endothelial cells [103]. Similarly, co-culture with other cell types such as pericytes or fibroblasts contributes to the formation of endothelial networks [104,105]. Still, in larger and more complex biofabricated constructs, strategies for spatial arrangement such as 3D bioprinting must be applied.

### 3.2. 3D Bioprinting

In biofabrication, 3D bioprinting is often used to achieve the precise spatial arrangement of cells in three-dimensional constructs, as well as to automate this process. It can also be used to create tubular structures that mimic the vasculature of native tissues. 3D printing, also known as additive manufacturing, refers to a process in which material is applied layer by layer to produce three-dimensional objects. As well as being utilized in the industrial sector, 3D printing is employed in a variety of other fields, such as medicine or research. Conventional acellular 3D printing is generally distinguished from bioprinting, in which living cells are directly printed.

Bioprinting involves the computer-aided, precise assembly of biological components such as cells, growth factors, and biomaterials, into tissue-mimicking constructs. This technique relies on biocompatible substances, usually biopolymer hydrogels, which not only support the printing process but also function as structural matrices for embedded cellular components [106].

**Figure 2:**
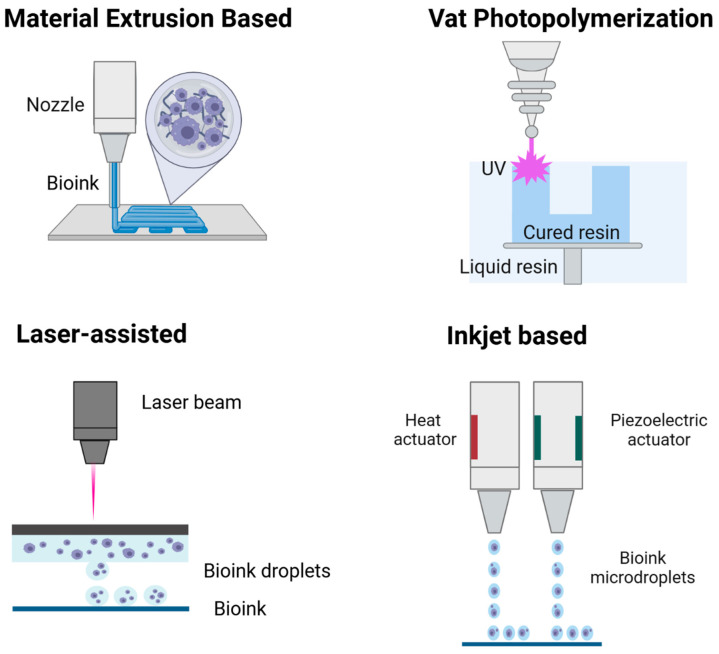
Overview of 3D bioprinting techniques. Schematics showing the basic principles of 3D bioprinting methods, such as material extrusion-based, laser-assisted, or inkjet-based printing, as well as vat photopolymerization. Created in BioRender. Sandor, E. (2026) https://BioRender.com/uet3xho (accessed on 5 July 2026).

The bioink is defined as the combination of cells and the printable scaffold material. In contrast to that, printable materials without cells are often referred to as biomaterial inks. The main technologies in bioprinting, as well as the biomaterials and bioinks, have been extensively reviewed elsewhere [6,107,108,109]. Here, we will give a short overview of the main technologies used in the 3D printing of vascular-like structures, which are also summarized in Figure 2.

Extrusion-based printing is frequently applied for the printing of tubular, blood vessel-like structures. It involves the controlled deposition of molten or highly viscous materials [110]. For the bioprinting of living cells, viscous materials such as hydrogels are extruded from a printing nozzle or needle via pneumatic or mechanical extrusion. This method enables the fabrication of cellular aggregates and scaffolds for soft tissues, offering fine-tuned control over porosity, geometry, and interconnectivity. However, challenges include viscosity constraints, limited resolution, and potential cellular damage from shear forces, cellular deformations, and elevated processing temperatures, as well as the extended time required to fabricate intricate structures [111]. Extrusion bioprinting has been successfully applied to print perfusable tubular structures containing endothelial cells, often in combination with other cell types such as MSCs or SMCs [112,113,114]. Cells in these constructs showed good survival and maturation. However, the long-term stability was impaired by degradation of the biomaterial.

In vat photopolymerization, a liquid photopolymer is selectively polymerized by visible or UV light [115]. The two main technologies are stereolithography and digital light processing (DLP). Stereolithography employs the sequential photopolymerization of biocompatible resins, activated by a high-precision laser beam [116,117]. In contrast, DLP uses a digital micro-mirror device (DMD) to crosslink an entire layer of the resin layer at once. DLP printers can therefore often print faster than SLA 3D printers, while stereolithography printers tend to achieve slightly higher resolutions. Photo-crosslinkable hydrogels such as gelatin methacryloyl (GelMA), methacrylated hyaluronic acid (HAMA) or methacryloyl-modified silk (Sil-MA) are compatible with a variety of cell types and can be printed with high precision. The high resolution of SLA has been applied to print hydrogel scaffolds with complex channel networks [118,119]. With advanced technologies such as two-photon photopolymerization, channels in the microvascular range of 10–30 µm have been obtained [120].

In inkjet bioprinting, droplets of bioink in the picolitre range are selectively deposited onto a substrate. In thermal inkjet printing, a heat actuator is used to generate heat bubbles leading to the formation of droplets at the nozzle, while piezoelectric inkjet printing utilizes piezoelectric ceramics to generate droplets [121,122]. One of its key advantages is the absence of a nozzle, which accommodates a diverse range of bioink viscosities while circumventing the clogging issues commonly associated with extrusion-based methods. Therefore, high-resolution tissue models can be generated. Still, the limitations of this method include reduced cell survival under excessive thermal and mechanical stress, as well as nozzle blockage due to cellular aggregation, which leads to lower cell densities within printed constructs [123]. Several groups have demonstrated the layer-by-layer printing of bifurcated vascular-like structures, both with and without cells [124,125].

Laser-assisted bioprinting utilizes a laser beam to capture and deposit cells or transfer material from a donor film onto a receiving substrate through a process known as pulsed laser-induced forward transfer (LIFT) [126,127]. Its high printing accuracy and resolution make this technique particularly suited for multilayer cell patterning and in situ bioprinting [128,129,130]. Furthermore, the mechanical stress on the cells is lower than with other printing methods, resulting in high viability rates after printing [126,131,132]. However, this method requires expensive equipment and materials, which limits its use for smaller laboratories.

### 3.3. Sacrificial Materials

Sacrificial materials can be used to create hollow structures or provide physical support. The sacrificial material can be printed alongside a non-sacrificial hydrogel as a physical support for hollow structures [133]. Alternatively, the sacrificial material can be directly incorporated into the hydrogel matrix in order to introduce porosity into the resulting construct. After the bioink is crosslinked, the sacrificial material is removed, thus leaving an internal void structure. Common sacrificial materials include gelatin, Pluronic, polyvinyl alcohol (PVA) or alginate. The removal mechanism depends on the physical and chemical properties of the sacrificial material: gelatin is a temperature-sensitive material that forms an aqueous solution at 37 °C but turns into a gel state below 30 °C [134]. Consequently, it can be removed from the constructs by elevating the temperature above 37 °C. Similarly, Pluronic forms a gel above 20 °C, but liquefies at 4 °C [135]. Water-soluble materials such as PVA or Pluronic can be dissolved in an aqueous solution [136,137]. Ionically crosslinked materials such as alginate are removed by the addition of a chelating agent [138].

A simple approach to facilitate diffusion in larger constructs is the introduction of pores. The porosity of a hydrogel scaffold can be increased by the addition of a porogen, such as sacrificial microparticles. Higher porosity enhances oxygen diffusion through the matrix and can also improve the self-organization of embedded endothelial cells into microvascular-like structures [139,140,141]. Alternatively, foaming hydrogels can be obtained by the controlled formation of air bubbles [142].

Also, constructs with perfusable microchannels can be 3D printed to simulate a vascular network. To prevent these channels from collapsing, they may initially be filled with a sacrificial material until the surrounding hydrogel matrix is crosslinked [136]. Advanced printing methods such as two-photon polymerization enable the generation of microvascular-like structures with high resolution [120].

### 3.4. Assessing Angiogenesis In Vitro

For biofabricated constructs intended for subsequent implantation in vivo, several angiogenesis assays can be performed to determine the angiogenic potential of the biomaterial and exclude potential negative effects on vascular cells. Angiogenesis assays are also used to determine the optimal concentration of pro-angiogenic substances added to the matrix. Colorimetric metabolic activity assays such as the WST-8 assay can be performed to determine the viability of endothelial cells inside the constructs [143]. This assay can be used to exclude negative effects on cell survival and proliferation, but it has limited specificity.

Another common approach is the spheroid sprouting assay, for which endothelial cell spheroids are embedded in the hydrogel matrix. The sprout number, sprout length and the migration of cells in the hydrogel are then determined [144]. Automating the imaging and analysis also enables high-throughput screening [145]. However, the results of this assay can vary between cast and printed hydrogels [146].

The tube formation assay is primarily used to measure the formation of capillary-like structures by endothelial cells seeded on a basement membrane [147]. Pro- or anti-angiogenic substances can be added to the culture medium, and the number of tubes and branch sites, as well as the tube length are measured. This assay can also be used for endothelial cells embedded in a hydrogel [148].

Another angiogenesis assay bridging the gap between in vitro and in vivo assays is the avian chorioallantoic membrane (CAM) model, which will be described in the next section.

## 4. The Chorioallantoic Membrane Model

**Figure 3:**
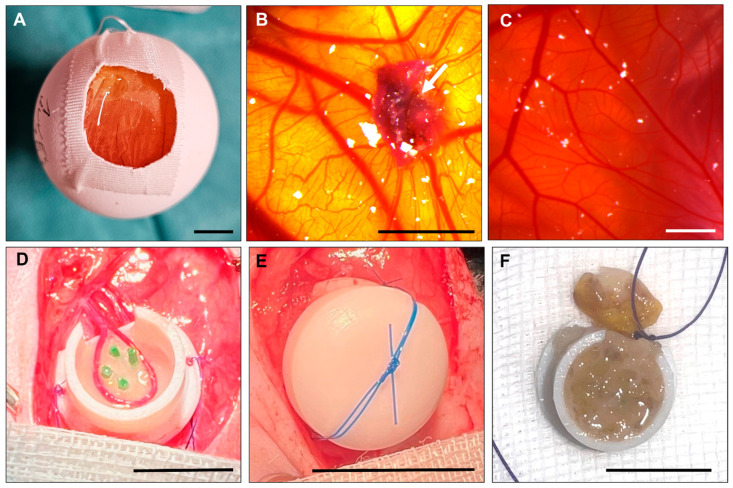
*In ovo* and in vivo vascularization of hydrogel scaffolds. *In ovo* and in vivo vascularization of hydrogel scaffolds. (**A**) Macroscopic appearance of a fenestrated chicken egg used for the chorioallantoic membrane (CAM) model. (**B**) Bioprinted scaffold placed on the CAM. (**C**) Vascular network of the CAM at embryonic day 14. (**D**) Rat arteriovenous (AV) loop placed in an implantation chamber filled with hydrogel matrix. (**E**) Closed implantation chamber. (**F**) Explanted AV loop construct after 4 weeks of implantation. Blood vessels were perfused with yellow contrasting agent for better visualization. Scale bar = 1 cm.

As an intermediate step between in vitro and in vivo models, the avian chorioallantoic membrane (CAM) model is used (Figure 3A,B). In vitro assays typically include only one cell type and therefore cannot recapitulate many of the processes involved in angiogenesis. However, in vivo angiogenesis assays are often expensive, time-consuming, and they require approval for animal experimentation. In contrast to that, the CAM model provides a physiological environment that includes a rapidly developing, easily accessible vascular network, while avoiding many of the legal restrictions associated with animal experimentation. The CAM model is commonly used to test the effects of pro- or anti-angiogenic substances, but also harbors great potential in biofabrication for investigating the vascularization of biofabricated constructs.

The chorioallantois is a highly vascularized membrane, formed by the fusion of the mesodermal layers of the chorion and allantois of a developing avian embryo [149]. It provides nutrients to the embryo, enables gas exchange with the environment, and reabsorbs calcium from the eggshell, which is necessary for bone formation in the embryo. In the chick embryo, the allantois first appears at embryonic day (ED) 3 as an evagination of the endodermal hindgut [150]. From ED4 to 10, the allantoic vessels enlarge rapidly, and the mesodermal layers of the allantois fuse with the adjacent mesodermal layer of the chorion to form the chorioallantois. The development of the vascular system is completed at ED10, and the CAM is fully differentiated at ED13 [151]. The embryonic developmental period of the chicken is 21 days, with an experimental time window of about 7 days (ED7-14). Since a chick embryo is not considered a living animal until ED17, experiments using the CAM model usually do not require approval for animal experimentation [151]. After ED18, the immune system of the embryos is fully developed, which limits the implantation of xenogeneic cells [152]. It is assumed that chick embryos do not feel pain until ED14, although this has not yet been fully determined.

The embryos may be cultured *in ovo*, with the shell mostly intact, or *ex ovo* inside another container. For *in ovo* culture, eggs are rotated until ED3 to prevent the embryo from sticking to the shell membranes. A small window is cut into the shell to allow for experimental manipulation. This cultivation method is considered to provide a more physiological environment and generally results in a higher survival rate, but observation and the area for experimentation are limited [150]. For *ex ovo* culture, the contents of the egg are transferred to another container (usually a Petri dish) at ED3 or 4 [153]. This facilitates experimental manipulation and live imaging, but it usually results in a higher mortality rate among the embryos.

Although this model is most commonly used with chicken eggs, it can also be applied in other avian species such as quail, duck, or ostrich. Eggs of the Japanese quail (*Coturnix japonica*) have a smaller size and thinner shell compared to chicken eggs, which facilitates experimental manipulation and requires less space in the incubator [154]. *Ex ovo* cultivation can even be performed in 6-well plates. Compared to chickens, quails have a shorter developmental period of approximately 16.5 days. The experimental window is about 7 days (ED6-12) [155].

Larger bird species have slower embryonic development than chickens, which increases the experimental period. For example, the incubation time of the turkey (*Meleagris gallopavo*) or the mallard duck (*Anas platyrhynchos*) is about 26–28 days [151,156]. However, fertilized eggs of these species are often not readily available, and there is limited experimental data.

The ostrich (*Struthio camelus*) is the largest extant bird species with the largest CAM. Its developmental period of 42 days, as well as the size of its eggs (about 50 times larger than a chicken egg), offers new possibilities for experimental manipulation [157]. Although the availability of experimental data is still limited, the *in ovo* as well as *ex ovo* cultivation of ostrich embryos has been described [157]. The large surface area and longer incubation time are advantageous for studying tumor growth *in ovo*, since larger tumors can be obtained [158]. Furthermore, the size of ostrich embryos enables the use of routine imaging devices intended for examinations in humans, such as positron emission tomography (PET) devices, and allows for easier intravasal injections and blood sampling [158].

The CAM model was first used in 1912, when Murphy and Rous described the implantation of sarcoma cells into a developing chick embryo [159]. Today, the CAM model is used for the study of vascular development and angiogenesis, in cancer research, and for performing toxicity screenings.

In biofabrication, this model has gained attention for its potential to test the biocompatibility, angiogenetic potential, and scaffold integration of biomaterials [160,161]. To assess their pro- or anti-angiogenic properties, test substances can be either directly applied onto the CAM or incorporated into hydrogel matrices. The number of blood vessels and branches can be quantified by manual counting and measurement. Alternatively, digital image analysis tools such as ImageJ, Matlab, or CellProfiler, can be used [153]. The expression of pro-angiogenic genes can be compared using quantitative PCR [162]. The development of the CAM vasculature over a longer observation period can be monitored via intravital microscopy [163]. However, this method is mostly suited for *ex ovo*-cultivated embryos as the field of observation is larger. Tumor growth and vascularization may also be visualized via ultrasonography, bioluminescence or PET/MRI imaging, although these methods require specialized equipment [164,165,166].

In cancer research, tumor cells are grafted onto the CAM, either in suspension or embedded in a hydrogel matrix. The CAM model is widely used to study cancer cell motility, invasion, and metastasis, as these mechanisms cannot be fully replicated in vitro [167]. Bioprinting technology can be applied to generate complex scaffolds, which are then placed on the CAM to study their interaction with blood vessels or the metastatic behavior of cancer cells. This was shown by Li et al., who investigated the interaction of breast cancer cells and endothelial cells with the CAM blood vessels [168].

The CAM model can also be used to investigate the vascularization of biofabricated scaffolds. Kim et al. implanted nanocomposite-based scaffolds onto the CAM and were able to compare the degree of vascularization between different scaffold compositions [169]. Likewise, De Moor et al. demonstrated that pre-vascularized spheroids embedded in a gelatin-based matrix showed anastomosis with the CAM vasculature [170]. Furthermore, the effect of commonly used additives such as bioactive glass particles can be evaluated. For example, Moll et al. demonstrated that the addition of mesoporous bioactive glass particles increased the vessel density of the CAM [171]. Since the CAM model allows for larger sample sizes and shorter experimentation periods compared to in vivo rodent models, it can be used to optimize the concentration and composition of additives. The results of the CAM assay can then be further verified in other animal models.

## 5. In Vivo Vascularization of Biofabricated Constructs

When biofabricated constructs are to be implanted into an animal model, fast integration into the host vasculature is crucial for the successful engraftment and survival of the implanted cells. Consequently, several strategies have been developed to enhance vascularization of the constructs.

3D-printed tubular structures can enhance the neo-vascularization of implanted scaffolds in vivo. Son et al. used a bioink containing endothelial cells and adipose-derived stem cells (ADSC) for the bioprinting of microvascular patterns, which enhanced graft vascularization in a mouse model [172]. To further accelerate the recruitment of blood vessels, constructs can be supplemented with pro-angiogenetic growth factors such as vascular endothelial growth factor (VEGF), basic fibroblast growth factor (bFGF) or platelet-derived growth factor (PDGF). Chen et al. incorporated VEGF into a 3D-printed scaffold for sustained release to improve vascularization after subcutaneous implantation into rats [173]. Likewise, Han et al. developed vascular grafts supplemented with VEGF and PDGF to be used for blood vessel reconstruction [174].

Alternatively, constructs can be supplemented with endothelial cells, which then undergo remodeling into microvessels. This was shown by Schechner et al., who demonstrated that HUVECs overexpressing the survival gene Bcl-2 were able to form perfused vascular structures after implantation into a mouse model [175].

Constructs can also be pre-vascularized by implanting them into a highly vascularized area of the body. During several weeks of implantation, host blood vessels can grow into the construct. The pre-vascularized scaffold is then excised and transferred to the defect site. For example, Xu et al. pre-implanted scaffolds for bone regeneration into a muscle pouch, while Li et al. pre-vascularized scaffolds for nerve regeneration via subcutaneous implantation [176,177].

Another approach in animal models is the induction of axial vascularization, either by using the vascular bundle or the arteriovenous (AV) loop model. For the vascular bundle, an artery and a vein are dissected from the surrounding tissue and placed within a scaffold or an implantation chamber, thus providing intrinsic vascularization [91,178]. Kawai et al. used this method in a model of a critical-size bone defect in the rat [179]. They combined a 3D-printed scaffold with a vascular bundle comprising the epigastric artery and vein. Supplementing the scaffold with the pro-angiogenic and pro-osteogenic bone morphogenic protein 9 (BMP-9) further improved bone volume and union in the defect site. Similarly, Yang et al. demonstrated that the vascularization of a hydrogel scaffold with a vascular bundle can be increased via the supplementation with adipose-derived stem cells [180].

Although the vascular bundle is simpler to construct operatively, the AV loop model shows higher levels of angiogenesis and tissue generation [178]. Because of its potential for the generation of axially vascularized tissue, the AV loop model will be further described in the following section.

### The Arteriovenous (AV) Loop Model

The rat AV loop model was first described by Erol and Spira in 1980, who observed the formation of a new capillary bed originating from an arteriovenous fistula (Figure 3C,D) [181]. Using an operating microscope, the femoral artery and vein of a rat are microsurgically prepared [182]. An interpositional vein graft (IVG) is then taken from the contralateral leg to ensure a sufficient length of the loop. The IVG is anastomosed between the femoral artery and vein using single interrupted sutures. The AV loop is then placed in an implantation chamber, which can be filled with a hydrogel matrix and cells. Neovascular sprouting then occurs, originating from the loop, particularly from the venous parts. As a result of increased endovascular pressure and shear stress, the vein graft undergoes an arterialization process, leading to an enhanced angiogenic response [183].

Since the blood vessels have a diameter of less than 1 mm, this step requires microsurgical expertise. A retrospective study by Polykandriotis et al. analyzing the outcome of 612 rat AV loop surgeries found a significant correlation between surgical experience and practice and the outcome of the surgeries [184]. Higher levels of experience result in a shorter duration of the surgeries and lower thrombosis rates, thus reducing the number of animals excluded from the analysis due to thrombosis of the loop vessels.

The implantation chamber can either be perforated or closed: perforated chambers allow additional extrinsic vascularization from the periphery, resulting in a higher degree of vascularization [185]. In contrast to that, closed chambers are only vascularized via the AV loop, which reduces the influence of external factors on the implanted construct. Hessenauer et al. also used an observation chamber for intravital microscopy, enabling the visualization of the growing vasculature during the implantation period [186]. Though the implantation chamber is typically filled with a hydrogel matrix, Mian et al. demonstrated the formation of new tissue originating from the AV loop even in the absence of an added matrix [187].

Although this model is predominantly used in the rat, it has also been applied in other animals such as rabbits, sheep or goats. Because of their larger size, they can be used to generate constructs of a more clinically relevant size. Dong et al. described an AV loop model in the rabbit, in which the popliteal artery was anastomosed with the femoral vein, which eliminates the need for an IVG [188]. With this model, they were able to obtain a vascularized bone substitute of 6 × 8 × 10 mm^3^. Likewise, Eweida et al. implanted a scaffold of 2 × 3 cm^2^ into a mandibular defect model of the goat, using the facial vessels to create an AV loop [189]. Beier et al. also described an AV loop model in the sheep, in which the saphenous artery and vein were anastomosed [190]. This was used to generate vascularized bone tissue with a total volume of 16 cm^3^ for a long bone defect model. Similarly, Kengelbach-Weigand et al. used the sheep AV loop model for the generation of bone substitute tissue supplemented with autologous MSCs and EPCs [191].

The AV loop model can be applied to investigate the vascularization of different biomaterials. Steiner et al. compared the vascularization of electro-spun and wet-spun recombinant spider silk matrices, which showed that the thinner electro-spun fibers resulted in faster biodegradation and higher levels of vascularization [94]. A study by the same group demonstrated that the modification of spider silk proteins with the RGD tag enhances vascularization [192]. Likewise, Heltmann-Meyer et al. investigated the vascularization, degradation, and biocompatibility of GelMA and ADA-GEL hydrogels [193,194].

Another application of the AV loop model is the generation of bioartificial tissue. Several studies report the formation of bone tissue upon the implantation of bone substitutes and osteogenic or endothelial cells. Arkudas et al. utilized a bovine cancellous bone matrix supplemented with osteoblasts for implantation in the rat AV loop model [195]. The survival of the osteoblasts was significantly higher than in the subcutaneously implanted control group. However, an intense foreign body reaction to the matrix was observed, which highlights the importance of an in vivo model to assess biocompatibility. Bone formation in the AV loop model was further improved by Buehrer et al., who supplemented the bone substitute matrix with MSCs and BMP-2 [196]. The importance of the vascular supply was demonstrated by de Silva et al., who showed that intrinsic vascularization by an AV loop significantly improved bone formation compared to the control group [197]. The therapeutic potential for the treatment of bone defects was shown by Arkudas et al. in a critical-size femur defect model [198].

Tee et al. used the AV loop model to generate cardiac muscle flaps [199]. Cardiomyocytes implanted into the AV loop chamber formed a muscle flap showing spontaneous contractions, which was then transplanted into recipient rats. Although no direct contact between the host myocardium and the flap’s cardiac muscle was observed, the cardiomyocytes remained viable throughout the experimental period of 4 weeks.

For the generation of skeletal muscle tissue, Bach et al. implanted myoblasts into a fibrin matrix which had been pre-vascularized by an AV loop [200]. Even though the myoblasts survived and retained their myogenic phenotype, no formation of skeletal muscle-like tissue was observed.

Since nervous stimulation is crucial for the development of functional muscle tissue, a neurotized variation called the EPI loop has been developed [201]. In this model, the saphenous artery and superficial inferior epigastric vein, together with a motor nerve branch are used to induce the myogenic differentiation of implanted cells. However, the use of these vessels results in an unfavorable entry angle of the saphenous artery. Consequently, the EPI loop model was modified by Kratzer et al., who instead used the superficial interior epigastric artery [202]. Xu et al. utilized this modified EPI loop model to generate vascularized and neurotized skeletal muscle tissue by implanting myoblasts and adipogenic MSCs onto nanofiber scaffolds [203].

The AV loop model can also be used to investigate the characteristics of cancer, such as proliferation, metastasis, tumor dormancy and tumor–stromal interactions, in an isolated implantation chamber within a living organism. An et al. established a model of breast cancer, using different matrix materials such as alginate, fibrin, and polycaprolactone [204]. The influence of the hydrogel matrix was further highlighted by Schmid et al., who established a melanoma model representing different tumor stages [205]. Melanoma cells implanted in Matrigel or alginate/gelatin/hyaluronic acid displayed high proliferation rates, while cells in the commercially available Cellink Bioink entered a state of dormancy.

The implantation matrix can also be 3D-printed to achieve better control over the spatial structure of the construct. Weinhold et al. 3D-printed the lower half of a recombinant spider silk matrix with a porous structure, while casting the upper part [206]. Interestingly, angiogenesis occurred predominantly in the area of the cast hydrogel,. Also, Sandor et al. compared two different print designs for the implantation of ADA-GEL constructs supplemented with melanoma cells, which showed differences in tumor growth and fibrovascular tissue formation [207]. This was most likely caused by the swelling behavior of the ADA-GEL matrix, as size extension of the construct can impair the blood flow through the AV loop if the construct design is not adjusted to accommodate for swelling. These results demonstrate that print design and material behavior have to be taken into account in sensitive experimental systems such as the AV loop model.

## 6. Application of Vascularized Biofabricated Constructs in Advanced Tissue Models

Biofabrication holds great potential for the creation of advanced in vitro tissue models to investigate organ function, disease modeling, and pharmacological studies. Cells in 2D culture systems often display differences in morphology, gene expression and treatment sensitivity compared to cells in 3D systems [208]. 2D culture systems cannot replicate fundamental aspects of the cellular in vivo environments, such as cell–cell and cell–matrix interactions or the spatiotemporal distribution of oxygen and nutrients [208,209,210]. Different bioprinting methods and biomaterials can be used to create tissue models that incorporate ECM components, tissue-specific cell types and vascular-like structures. However, the complexity of native tissues and organs still constitutes a major limitation. Not only are organs structured in a highly organized and hierarchical manner, but they consist of various specialized types of cells.

Different cell types often have specific requirements in terms of nutrients, growth factors, cell–cell and cell–matrix contacts in order to preserve their native phenotype [211]. Furthermore, the construct size is still limited by oxygen and nutrient diffusion due to a lack of vascularization. Consequently, many advanced tissue models have been modified to include perfusable channels or vascular cells to mimic the vascular system. These advanced models are of great interest for basic research, disease modeling, drug screening and personalized medicine. Still, biofabricated models currently only represent a limited part of the native organ.

The biofabrication of kidney models is of special interest due to the kidney’s role regarding drug elimination. Its complex structure, including the glomerular filtration barrier and the subsequent proximal and distal tubules, poses a challenge when generating models using 3D bioprinting and microfluidics. Biofabricated models therefore only replicate parts of the kidney. For example, Lin et al. created a model of the proximal tubules by seeding proximal tubule epithelial cells and vascular endothelial cells into 3D-printed tubular structures [212]. This model used tissue-specific cells to better recapitulate the native function of renal endothelial cells. Likewise, Singh et al. used decellularized kidney ECM and tubular epithelial cells for a vascularized model of the renal parenchyma, although HUVECs were used instead of renal endothelial cells [213].

Since the kidneys play a central role in drug metabolism and elimination, drug-induced nephrotoxicity is a major concern in screening for new drugs. Consequently, some bioprinted kidney models were specifically adapted for toxicity screening. King et al. developed a bioprinted proximal tubule model comprising renal fibroblasts, HUVECs, and tubule epithelial cells for toxicity screening [214,215]. This model displayed a dose-dependent decrease in tissue viability when treated with the nephrotoxin cisplatin, as well as a fibrotic response after treatment with TGF-β. A combined liver–kidney-on-a-chip model was developed by Huang et al. to evaluate both hepatic and renal toxicity [216]. They incorporated liver sinusoidal endothelial cells as well as HUVECs to introduce a vascular component.

Bioprinted liver models have been shown to produce liver-specific proteins and metabolites such as albumin, urea or lactate dehydrogenase [217,218,219]. Many advanced models include elements of the vasculature, for example via the incorporation of perfusable channels or the encapsulation of endothelial cells [218,220]. By using patient-derived cells, preclinical models of several hepatic diseases have been developed. Tan et al. investigated the role of different hepatic cell types in nonalcoholic steatohepatitis by printing chimeric tissue models comprising healthy and diseased cells, including liver sinusoidal endothelial cells [221]. Similarly, Norona et al. used bioprinted liver models to assess the role of fibrinogenic agents in the development of liver fibrosis, incorporating HUVECs [222,223].

In cardiac biofabrication, recent advances in bioprinting have made it possible to generate contractile heart models derived from cardiomyocytes. Various studies have reported bioprinted heart models that displayed synchronous contractions over several weeks and responded to pharmacological or electrical stimuli [224,225]. Some models have even used patient-derived cells to study congenital heart disease. For example, a study by Wolfe et al. used hiPSC-derived cardiomyocytes from patients with hypoplastic left heart syndrome to print cardiac tissue constructs in order to investigate the molecular mechanisms behind the disease [226]. These cardiac models can be further advanced by adding a vascular component. Zhang et al. established a bioprinted endothelialized myocardium-on-a-chip model [227]. They first printed hydrogel scaffolds containing endothelial cells, which migrated to the periphery to form a layer of confluent endothelium. Next, they seeded the scaffolds with cardiomyocytes to generate a myocardial structure capable of spontaneous contractions. The constructs were then embedded into a microfluidic perfusion bioreactor. Treatment with the anti-cancer drug doxorubicin resulted in a significant decrease in the beating rate, which shows the model’s potential as a drug screening platform for cardiotoxicity. Furthermore, Landau et al. demonstrated that the addition of macrophages into an endothelialized heart-on-chip model promotes the stabilization of microvessels formed by HUVECs [228].

The biofabrication of skin substitutes is of high clinical relevance for the treatment of large-scale skin defects due to the limited availability of autologous skin grafts [229]. The skin consists of three main layers: the epidermis, dermis, and hypodermis, which all contain different cell types. This layered structure must be taken into account when creating tissue models. For example, the epidermal and dermal layers can be replicated using keratinocytes and fibroblasts, respectively. The imitation of pigmentation and follicular layers, on the other hand, requires the incorporation of melanocytes and follicle dermal papilla cells. In biofabricated skin models, 3D printing is used for the spatial arrangement of different cell types to form distinct skin layers. Jorgensen et al. incorporated six cell types, including dermal microvascular endothelial cells, into a multilayered skin substitute [230]. This construct was shown to accelerate wound healing in a mouse model, where it integrated into the host vasculature. Similar results were obtained by Wang et al., who incorporated HUVECs into a double-layer conductive skin scaffold along with fibroblasts and keratinocytes [231].

Precise control over the spatial distribution of cells is also crucial for other vascularized tissue models. Song et al. recapitulated the layers of the outer blood–retina barrier by bioprinting iPSC-derived endothelial cells, pericytes and fibroblasts onto a scaffold and seeding retinal pigment epithelium cells on top [232]. Similarly, Wang et al. created a perfusable model of the blood–brain barrier containing astrocytes, pericytes, and immortalized brain endothelial cells [233]. A model of the alveolar barrier was established by Kang et al., who printed a three-layered model consisting of alveolar cells, lung fibroblasts and lung microvascular endothelial cells [234]. The recent advances in the biofabrication of various organ models and organ-on-a-chip systems have been reviewed in detail by, for example by Nunes et al., Monteduro et al. or Zhou et al. [235,236,237].

In cancer research, the role of the tumor microenvironment (TME) has increasingly come into focus. The TME consists of various stromal cell types such as cancer-associated fibroblasts, immune cells or mesenchymal cells, as well as blood vessels, signaling molecules and the ECM components [238]. Since the TME has a significant impact on tumor cell proliferation, metastasis, and treatment resistance, it is highly relevant in a clinical context. To recreate the tumor niche in vitro, various features of the TME have been incorporated into 3D-printed cancer models. For example, Maggiotto et al. introduced a vascular component to a neuroblastoma model by 3D printing channels lined with HUVECs, while Braham et al. incorporated MSCs and endothelial progenitor cells into printed scaffolds to recapitulate the endosteal and perivascular subniches in a myeloma model [239,240].

Cells in 2D culture often show higher sensitivity to drug treatment than cells in 3D culture [208,241]. As a result, numerous compounds show promising results in 2D drug screenings, but fail in subsequent in vivo studies [242]. In recent years, several 3D screening models have been developed. To identify potential new drugs from compound libraries, automated high-throughput screening is performed. These assays have predominantly been used in 2D cell culture. However, recent advances in the automated generation of multicellular tumor spheroids or organoids enable the biofabrication of 3D screening platforms [243,244,245]. Some of these models also include vascular cells [214,216,227]. To introduce components of the TME, other cell types such as fibroblasts, pancreatic stellate cells or immune cells may also be added [246,247,248]. As described previously, biofabricated models of various tissues such as the liver, heart or kidney have been adapted for drug screening [214,215,216,249]. For a further step towards personalized medicine, patient-derived cells can be incorporated to predict the treatment response [250,251,252].

## 7. Future Perspectives in Biofabrication

Vascularized biofabricated constructs show great potential in both in vitro and in vivo studies. By incorporating a vascular element, these constructs resemble the native environment more closely. However, the process of translating these technologies into clinical practice is still in its early stages. Although many biofabricated models show great success in vitro or in small animal models, clinical translation is often limited by the small size of the constructs. Since larger constructs have a limited supply of oxygen and nutrients, integration into the host vasculature is essential for graft survival. Also, most constructs lack critical functional elements such as innervation or other supporting cell types required for correct function in vivo. Furthermore, scalability and reproducibility are often restricted by the high complexity of biofabricated models. Many studies successfully generate millimeter-scale channels, but cannot recapitulate the hierarchical structure of arteries, capillaries and veins found in native tissues. However, a higher level of standardization may be achieved by incorporating process automation, sensor integration and quality assessment into the biofabrication workflow [253]. New advances in fields such as robotics and artificial intelligence may therefore contribute to better clinical translation. Multidisciplinary collaboration will thus play a major role in the future of biofabrication.

In contrast to small-molecule drugs, there is a relative lack of clinical experience with cellular therapy products [254]. Cellular therapies can persist in the patient’s body for an extended period of time or have an extended effect even if the therapy product is no longer present. Furthermore, cellular therapeutic products may require surgery or invasive methods of delivery, which pose an additional risk. Also, implanted cells can differentiate in vivo into undesired cell types, form tumors, or migrate from the implantation site. Prior to clinical trials, preclinical in vitro and in vivo proof-of-concept, pharmacology, and toxicology studies are performed. In early-phase clinical trials, dose explorations, as well as feasibility and activity assessments, are performed.

The integration into the host vasculature still poses a major challenge for the successful implantation of biofabricated constructs. Many encouraging studies have been performed in small animal models such as mice, rats or rabbits. However, these models possess shorter diffusion distances and smaller tissue volumes than those required in humans, and constructs often fail when scaled to human-sized tissue. Larger animal models such as sheep, goats or pigs can be used to generate clinical-sized constructs, but they require specially trained personnel and equipment, and they are very costly.

The clinical trial process and the approval procedure vary between different countries. In the USA, the Food and Drug Administration (FDA) regulates all medical products. Guidance documents regarding the development and approval process of cellular and gene therapy products are provided on the FDA website [254,255]. Within the European Union (EU), medicines can be authorized through one of two main routes: the centralized route or the national route. The European Medicines Agency (EMA) evaluates the applications for drug approval, and it provides guidelines for the non-clinical and clinical requirements for investigational advanced therapy medicinal products, as well as human cell-based products [256,257]. Information about national authorization procedures can be found in the national registers of authorized medicines [258]. In China, the China Food and Drug Administration (CFDA) is responsible for registering pharmaceutical products, while the Center for Drug Evaluation (CDE) is in charge of reviewing applications for drug registration [259,260]. The regulatory procedures in other Asian countries such as Japan, South Korea or India has been previously reviewed [261,262,263].

Even though biofabricated constructs are not yet applied in large-scale clinical trials, there have been some examples of the successful clinical translation of tissue engineering. The feasibility of tissue-engineered constructs has been assessed in several case reports, such as for vascular grafts, skin substitutes, or cardiac bioimplants [264,265,266]. Combining conventional tissue engineering methods with bioprinting technology and advanced pre-vascularization strategies could further improve the efficiency of implanted constructs.

Biofabricated tissue models are also employed to reduce the number of animals used for research purposes. The FDA Modernization Act 2.0 allows the use of human-relevant in vitro models as an alternative to animal testing to generate pre-clinical drug trial data [267]. Incorporating human or patient-derived cells into drug screening could make the process more efficient and improve the translation from bench to bedside.

The advancement of biofabrication, coupled with the ongoing enhancement of vascularization, offers promising solutions to longstanding challenges in tissue engineering. Although the translation of these technologies into clinical practice remains in its early stages, initial breakthroughs highlight the potential of bioprinting for precise cellular deposition and the incorporation of bioactive compounds. The effectiveness of these approaches hinges on multidisciplinary synergy, bringing together expertise from biomaterials research, cellular biology, biochemistry, engineering, and medical professionals specializing in plastic and reconstructive surgery.

## Figures and Tables

**Table 1:** Comparison of different in vitro, *in ovo* and in vivo strategies for the vascularization of biofabricated constructs.

Method	Advantages	Disadvantages
3D-Bioprinting	− Good reproducibility − Automation possible − High throughput − Comparatively low operating costs	− Lower complexity − Biomaterials must be printable − May reduce cell viability − Initially high cost for printers
Incorporation of vascular cells	− Good to moderate reproducibility − High to medium throughput (depending on cell type) − Possible to use patient-derived cells	− Lower complexity − Comparatively high costs for consumables (e.g., specialized cell culture medium) − Results may vary depending on isolation method or donor background
Chorioallantoic membrane (CAM) model	− Usually does not require approval for animal experimentation − Medium throughput − Higher physiological relevance	− Limited experimentation window − Fertilized eggs may not be readily available − Medium cost
In vivo prevascularization	− High physiological relevance − Higher clinical applicability	− Requires approval for animal experimentation − Requires surgical skills − Low throughput − High cost
In vivo axial vascularization (e.g., AV loop)	− High physiological relevance − Higher clinical applicability	− Requires approval for animal experimentation − Requires microsurgical skills − Low throughput − High cost

**Table 2:** Comparison of the most commonly used types of vascular cells in biofabrication.

Cell Type	Advantages	Disadvantages
Primary HUVECs	− Relatively easy to isolate and cultivate − Widely available	− Do not recapitulate organ-specific functions − Limited number of cell divisions
Immortalized HUVECs	− Easy to cultivate − Widely available − Unlimited cell divisions	− Gene expression different from primary cells − Do not recapitulate organ-specific functions
hiPSCs	− Can be differentiated into organ-specific cell types − Possible to use patient-derived cells	− Labor-intensive differentiation − High costs − Variation in gene expression depending on donor background
Specialized vascular cells (e.g smooth muscle cells, pericytes)	− Recapitulate organ-specific functions	− High levels of heterogeneity − Often difficult to isolate

## Data Availability

No new data were created or analyzed in this study.
